# Generation of Transgene-Free Semidwarf Maize Plants by Gene Editing of *Gibberellin-Oxidase20-3* Using CRISPR/Cas9

**DOI:** 10.3389/fpls.2020.01048

**Published:** 2020-07-09

**Authors:** Jiaojiao Zhang, Xiaofeng Zhang, Rongrong Chen, Li Yang, Kaijian Fan, Yan Liu, Guoying Wang, Zhenjing Ren, Yunjun Liu

**Affiliations:** Institute of Crop Sciences, Chinese Academy of Agricultural Sciences, Beijing, China

**Keywords:** CRISPR/Cas9, gene-editing, gibberellin oxidase, semidwarf, transgene-free

## Abstract

The “green revolution” gene gibberellin oxidase contributes to the semidwarf phenotype, improving product and lodging resistance. Dissecting the function of GA biosynthetic genes would be helpful for dwarf maize breeding. In this study, we edited the maize *GA20ox3* gene and generated semidwarf maize plants using CRISPR/Cas9 technology. Application of exogenous gibberellin can recover the dwarf phenotype, indicating that the mutants are gibberellin deficient. The contents of GA_12_ and GA_53_ were elevated in the mutants due to the disruption of GA20 oxidase, whereas the contents of other GA precursors (GA_15_, GA_24_, GA_9_, GA_44_, and GA_20_) were decreased in the mutants, and the accumulation of bioactive GA_1_ and GA_4_ was also decreased, contributing to the semidwarf phenotype. Transgene-free dwarf maize was selected from T_2_-generation plants and might be useful for maize breeding in the future.

## Introduction

Gibberellin (GA) is an important hormone in plants and plays essential roles in plant growth and development, including embryogenesis, seed germination, stem elongation, and flowering (Yamaguchi and Kamiya, 2000; Binenbaum et al., 2018). The biosynthesis of GAs is complex and involves multiple steps. The precursors of GAs are synthesized in plastids and then modified further in the endoplasmic reticulum and cytosol to produce bioactive GAs including GA_1_, GA_3_, GA_4_, and GA_7_ (Hedden and Phillips, 2000). Loss of function of the genes involved in the gibberellin biosynthesis pathway or signaling pathway would make plants dwarf, and these dwarf mutant plants can be divided into GA-sensitive and GA-insensitive types. GA-sensitive dwarf mutants are caused by a deficiency of GA biosynthesis-related genes and the application of exogenous gibberellin can recover the dwarf phenotype, whereas the dwarf phenotype of GA-insensitive mutant plants cannot be recovered by the application of exogenous gibberellin (Galbiati et al., 2002).

Semidwarf plants can greatly contribute to crop improvement, as reported for semidwarf “green revolution” rice (Sasaki et al., 2002) and wheat (Peng et al., 1999). In “green revolution” wheat, orthologues of the Arabidopsis *Gibberellin Insensitive* (*GAI*) gene, which plays roles in GA signaling, are mutated. However, the phenotype of “green revolution” rice results from the mutation of *GA20ox-2*, which is involved in GA biosynthesis (Sasaki et al., 2002). For maize, *Dwarf8* gene has been identified as an orthologue of the *GAI* gene and been targeted by selection (Peng et al., 1999; Andersen et al., 2005). Other numerous dwarf maize mutants have also been reported (Bensen et al., 1995; Winkler and Helentjaris, 1995; Lawit et al., 2010; Jiang et al., 2012; Chen et al., 2014), but none of them can play the same essential roles as “green revolution” rice or wheat.

Overexpression or suppression of GA biosynthesis-related genes affects plant height and biomass through increasing the accumulation of bioactive GAs. For example, overexpression of *GA20ox-1*, which is an essential gene in GA biosynthesis, increased the stem biomass yield in poplar and tobacco plants (Eriksson et al., 2000; Biemelt et al., 2004). The ectopic expression of Arabidopsis *GA20ox* in transgenic tobacco increased bioactive GA levels and stimulated the growth plants (Sophia et al., 2004). Transgenic citrus plants overexpressing *GA20ox* presented higher active GA_1_ levels and increased plant growth (Fagoaga et al., 2007). Overexpression of *GA20ox* in Arabidopsis increased GA_4_ levels and seedling hypocotyl length (Coles et al., 2010). Ectopic expression of *GA20ox1* in maize increased endogenous GA levels and led to longer stems of the transgenic maize (Nelissen et al., 2012). Further research revealed that *GA20ox1* maize plants exhibited increased vegetative biomass compared with control plants (Voorend et al., 2016). Overexpression of the *CrGA20ox1* gene in *Camellia reticulata* improved vegetative growth; however, the suppression of the *CrGA20ox1* gene led to dwarf plants (Wang et al., 2018).

Recently, the clustered regularly interspaced short palindromic repeats (CRISPR)/Cas9 system has been applied for gene targeting in various species, including rice, wheat, cotton, Arabidopsis, tomato, and soybean (Li et al., 2013; Gao et al., 2017; Jacobs et al., 2017; Cai et al., 2018; Zhang et al., 2018a; Zhang et al., 2018b). Using the CRISPR/Cas9 system, the tomato *PROCERA* gene was edited to generate dominant or semidominant dwarf tomato mutants (Tomlinson et al., 2019; Zhu et al., 2019). A number of studies have reported the application of CRISPR/Cas9 in gene targeting in maize (Feng et al., 2016; Shi et al., 2017; Chen et al., 2018). However, there are no reports of the gene editing of GA biosynthesis-related genes for the generation of dwarf or semidwarf maize plants. The functions of the genes involved in GA biosynthesis in maize require further investigation.

Maize is an important crop in the world, and maize height is related to the architecture, lodging resistance, and grain yield of this crop. To increase maize yield, plant density has generally been increased (Duvick et al., 2010). However, a high plant density will lead to a risk of lodging. To avoid lodging, one breeding strategy is to moderately decrease maize height. In this study, we chose *ZmGA20ox3* as the target gene and used the CRISPR/Cas9 system to specifically induce targeted mutations of the *ZmGA20ox3* gene. Gene-edited “transgene-free” dwarf maize plants were generated, which might be useful for maize breeding in the future.

## Results

### Targeted Mutagenesis of *ZmGA20ox3* Using the CRISPR/Cas9 System

**Figure 1:**
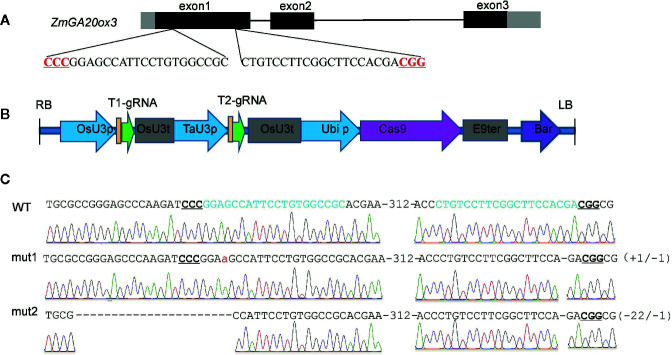
CRISPR/Cas9-mediated mutations at *ZmGA20ox3* target sites in plants. **(A)** Schematic illustration of the target sites. The black and gray boxes indicate the exons and UTRs, respectively, of the *ZmGA20ox3* gene. The black DNA sequence is the target sequence. Nucleotides in red represent PAM sequences. PAM, protospacer-adjacent motif. **(B)** The T-DNA region of the pBUE411-2gR-GA plasmid. **(C)** Sequence peaks of wild-type and representative homozygous mutants at target sites. The red lowercase letter represents an adenine insertion in mut1.

The CRISPR/Cas9-mediated genome-editing tool was utilized to edit the endogenous maize gene *ZmGA20ox3* (GRMZM2G368411). Two target sites were designed in the first exon of *ZmGA20ox3* (**Figure 1A**), and the corresponding sequence was synthesized and ligated into the pBUE411-2gR plasmid (Xing et al., 2014) to construct the pBUE411-2gR-GA vector (**Figure 1B**). The vector was transformed into the maize inbred line Cal *via* the *Agrobacterium*-mediated transformation method, and ten independent T_0_ transgenic lines were generated. The fragments including the target sites were PCR amplified from the transgenic plants and sequenced directly. The sequencing results showed that three heterozygous transgenic lines exhibited mutations at the target sites of the *ZmGA20ox3* gene. Because these T_0_ transgenic plants could not be self-pollinated in the greenhouse, they were crossed with the maize inbred line Cal to produce the T_1_ generation, which was then self-pollinated to produce T_2_ progeny.

Two types of homozygous mutant plants were chosen for further experiments. One was designated mut1, which presented an adenine insertion at the first target site and a cytosine deletion at the second target site; the other was designated mut2 and exhibited a twenty-two-nucleotide deletion at the first target site and a cytosine deletion at the second target site (**Figure 1C**). Both of these mutation types might result in a nonfunctional GA20ox3 protein.

### The Semidwarf Phenotype of Gene-Edited Plants

**Figure 2:**
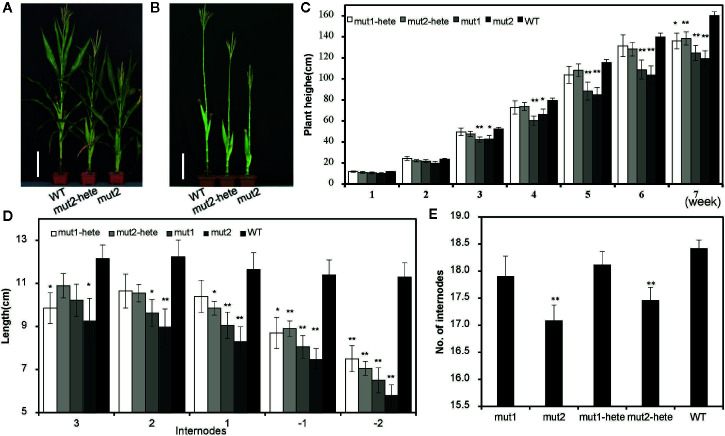
Plant height and internodes of the mutant plants. **(A, B)** Plant height of mut2 and wild-type plants at the mature stage. Plant leaves were stripped **(B)** or not **(A)**. Scale bar = 25 cm. **(C)** Plant height of mutant plants at different development stages. The values shown are the average ± S. E. for independent plants (number of mut1-hete =11, number of mut2-hete = 18, number of mut1 = 13, number of mut2 = 12, number of WT = 68) **(D)** The internode lengths of the mutant and WT plants. The internodes above the ear are labeled 1, 2, and 3, and the internodes below the ear are labeled -1 and -2. The values shown are the average ± S. E. for independent plants (number of mut1-hete =10, number of mut2-hete = 10, number of mut1 = 9, number of mut2 = 19, number of WT = 10). **(E)** The numbers of internodes in mutant and WT plants. Error bars represent the standard errors of nine biological repeats. The values shown are the average ± S. E. for independent plants (number of mut1-hete =10, number of mut2-hete = 10, number of mut1 = 9, number of mut2 = 9, number of WT =10). * indicates a significant difference at the *P* < 0.05 level, and ** indicates a significant difference at the *P* < 0.01 level.

The T_2_ generation plants of these two mutants showed an intermediate phenotype between WT and homozygous mutant plants (**Figures 2A, B**). We measured the plant height (PHT) of the mutant and WT plants once a week after the emergence of seedlings in the field. The PHTs of homozygous mut1 and mut2 plants were significantly shorter than that of the WT from the third week after emergence, while the PHTs of the heterozygous plants (mut1-hete and mut2-hete) were not significantly shorter than that of the WT until after the seventh week (**Figure 2C**). We also measured the -1 and -2 internodes under the ear and internodes 1 to 3 above the ear and found that most of the mutant internodes were markedly shorter than those of the WT (**Figure 2D**). The mut2 plants presented fewer internodes than the WT, whereas the mut1 internode numbers were similar to those of the WT (**Figure 2E**). We speculate that the reduced numbers and lengths of the internodes in the mutant plants might contribute to the dwarf phenotype.

### Morphological Comparison of Intersegmental Cells

**Figure 3:**
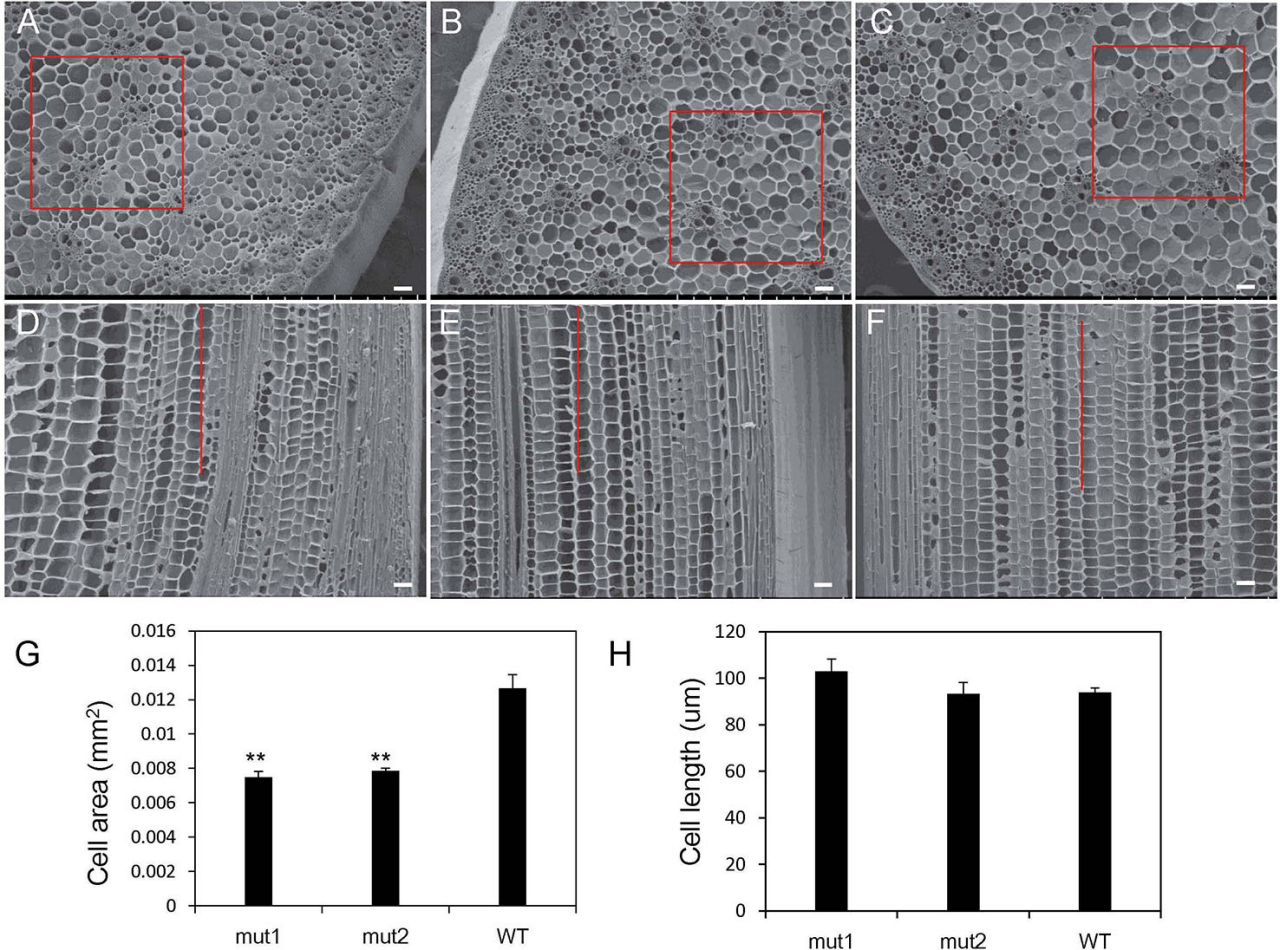
Intercellular morphology of *ga20ox3* mutant and WT plants. **(A–C)** showed the cross-section morphology of mut1 **(A)**, mut2 **(B),** and WT **(C)** plants. **(D–F)** showed the longitudinal sections of mut1 **(D)**, mut2 **(E)**, and WT **(F)** plants. **(G)** Cell area in a cross-section of the parenchyma. The cell numbers within the 0.81 mm^2^ area (the square box in **A–C**) were counted and the area of each cell was calculated by the formula: 0.81 mm^2^/cell numbers. **(H)** Cell length in a longitudinal section of the parenchyma. The cell numbers within 1 mm distance (the lines in **D–F**) were counted and the cell length was calculated by the formula: 1 mm/cell numbers. scale bar=0.1 mm; ** indicates significant differences at the *P<*0.01 level; error bars represent the SE of the mean (n_mut1_ = 6, n_mut2_ = 6, n_WT_ = 6).

To further explain the cytological basis of internode shortening in the *ga20ox3* dwarf mutant, the morphology of the internodes at the spike position in 12-week-old mut1, mut2, and WT plants grown in a greenhouse was observed by scanning electron microscopy. The cell area and cell length at the same location in the cross section (the square box in **Figures 3A–C**) or the longitudinal section (the lines in **Figures 3D–F**) were calculated. The results showed that the arrangement of vascular bundles, sclerenchyma cells, and parenchyma cells in both mutants and WT plants was regular, but the cross-sectional area of the mutant cells was significantly smaller than that of the WT (**Figures 3A–C**). The cell area of the mutant parenchyma was significantly different from that of the WT (**Figure 3G**). The length of the mutant cells did not differ from that of the wild-type cells (**Figures 3D–F, H**). These results suggest that the shortening of mut1 and mut2 internodes was not caused by a decrease in cell longitudinal length but by the decrease in cell numbers.

### GA Biosynthetic Pathway Was Affected in Gene-Edited Dwarf Maize Plants

**Figure 4:**
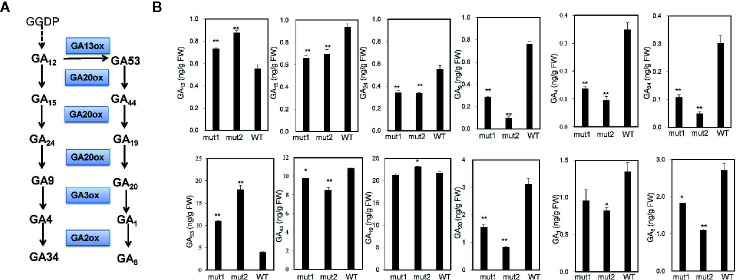
GA contents in mutant and WT plants. **(A)** Diagram of the GA synthesis pathway. **(B)** Endogenous contents of GA_12_, GA_15_, GA_24_, GA_9_, GA_4_, GA_34_, GA_53_, GA_44_, GA_19_, GA_20_, GA_1_, and GA_8_ in the stem apices of 7-week-old plants. Experiments were repeated with three biological replicates. * indicates significant differences at *P<*0.05 level; ** indicates significant differences at the *P<*0.01 level.

**Figure 5:**
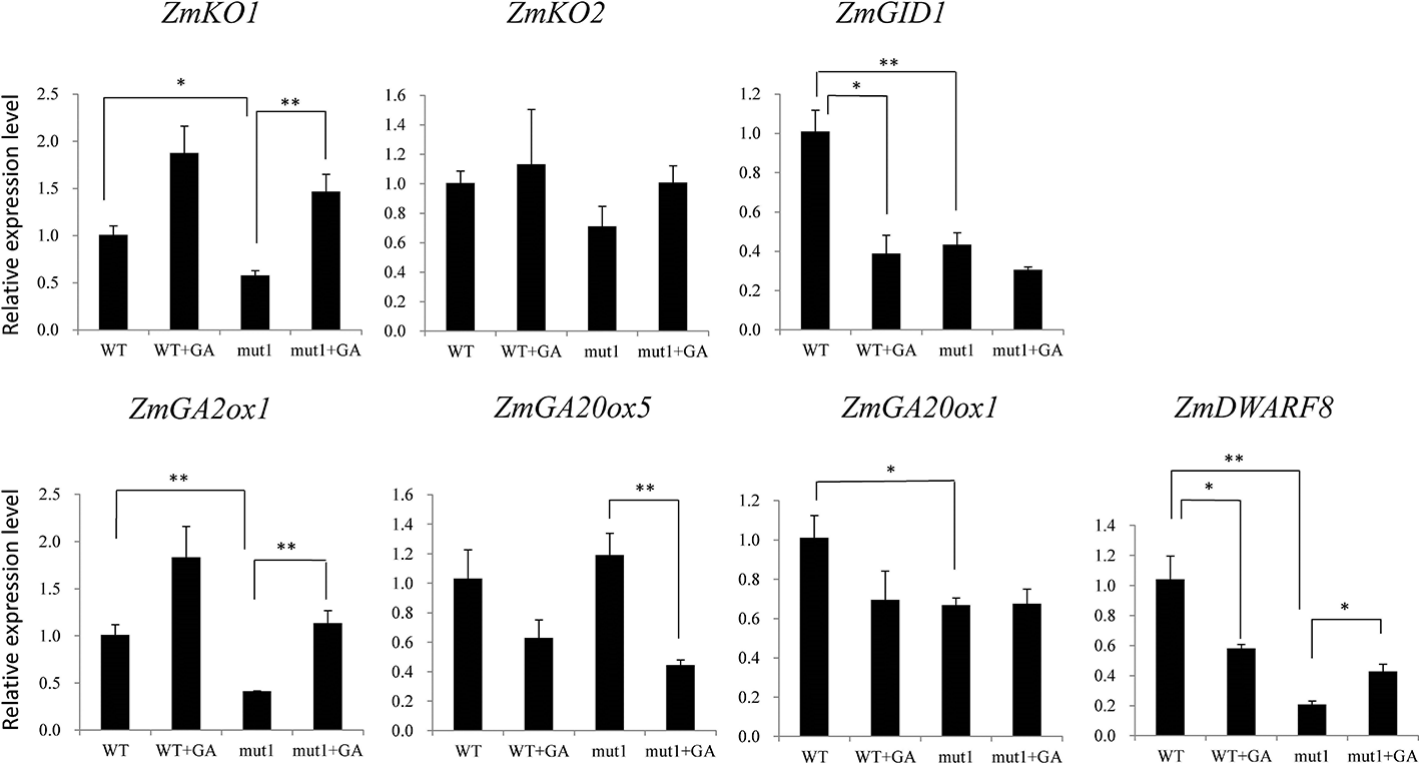
Effect of exogenous GA_3_ on gene expression levels in WT and mutant plants. Two week after seedling emergence, the plants were sprayed with 100 mg/L GA_3_ one times per day. Shoot samples were collected one week after treatment. Data was shown as average ± S.E. of three independent experiments. Experimental data was tested by student t-test analysis. * and ** indicate significant differences at the *P*<0.05 and *P*<0.01 level, respectively.

To determine whether the phenotype of the *ga20ox3* dwarfing mutant was caused by blocked gibberellin synthesis, we determined the contents of endogenous gibberellins in homozygous mutants and WT plants grown in a greenhouse. The results showed that the contents of GA_12_ and GA_53_ were elevated in the mutants due to the disruption of GA20 oxidase. However, the contents of other GA precursors (GA_15_, GA_24_, GA_9_, GA_44_, and GA_20_) were decreased in the mutants. The accumulation of bioactive GA_1_ and GA_4_ was also decreased in the mutants (**Figure 4**), which contributed to the semidwarf phenotype. Bioactive GA_3_ was not affected in the mutants. The decrease in the active endogenous gibberellin content in the mutant resulted in plant height dwarfing, indicating that the mutants belonged to the GA-deficient dwarfing mutant type. The expression levels of the GA biosynthetic pathway-related genes, i.e., *ZmKO1*, *ZmGID1*, *ZmGA2ox1*, *ZmGA20ox1*, were decreased in mut1 shoots (**Figure 5**), confirming the defective of GA biosynthetic pathway.

**Figure 6:**
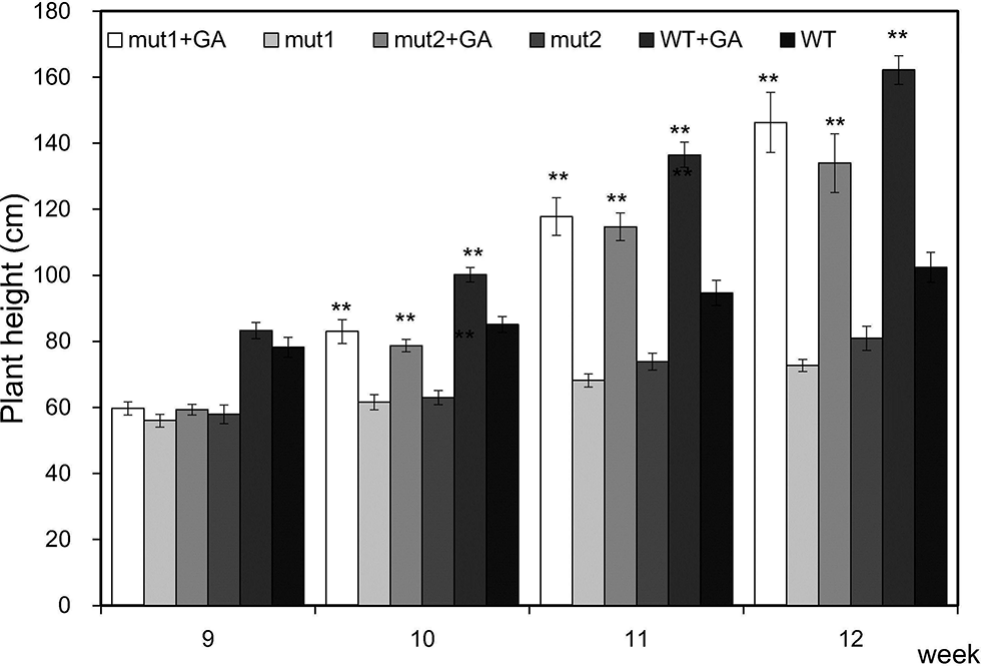
Effect of exogenous GA_3_ on the plant height of WT and mutants. Plant heights were measured 9 weeks after emergence. The values presented are the means ± S.E.s of 10 individual plants. ** indicates significant differences at the *P<*0.01 level.

To verify that the mutants are gibberellin deficient, we analyzed the phenotypic changes in the mutant when exogenous GA_3_ was applied. GA_3_ increased the PHT of the WT and mutant plants. After one week of treatment, the PHT of treated mutants was similar to that of untreated WT plants, indicating that exogenous GA_3_ could alleviate the difference in plant height between the mutants and the WT. With an longer treatment, the PHT became significantly higher (**Figure 6**). The results showed that the signal transduction pathway of gibberellin in the *ga20ox3* mutant was functional and could respond to exogenous GA_3_ treatment and restore plant height, indicating that the mutants are gibberellin deficient.

For WT plants, GA_3_ application downregulated the expression of *ZmGID1*. For mutant plants, GA_3_ application upregulated the expression of *ZmKO1* and *ZmGA2ox1*, but downregulated the expression of *ZmGA20ox5*. *ZmDWARF8*, which encodes DELLA protein and participate in GA signaling (Lawit et al., 2010), was downregulated by GA_3_ application in WT plants, whereas was upregulated by GA_3_ application in mutant plants (**Figure 5**).

### Generation of Transgene-Free Dwarf Maize Plants

**Figure 7:**
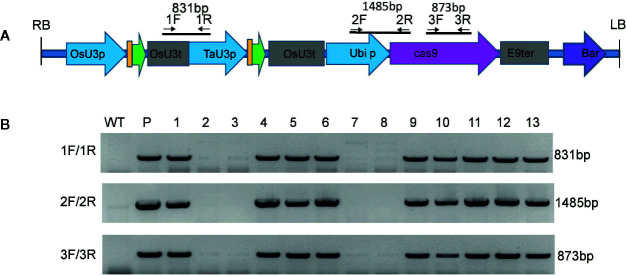
Screening of transgene-free *ga20ox3* mutants. **(A)** Schematic representation of the T-DNA region of the pBUE411-2gR-GA vector and the locations of the primers. **(B)** Agarose gel electrophoresis of three PCR fragments of T-DNA. WT, DNA of wild-type plants; P, plasmid pBUE411-2Gr; 1–13, individual T_2_ plants.

To generate transgene-free dwarf plants, the T_2_ generation dwarf plants from the mut1 line were screened by PCR amplification of the *OsU3* terminator, *TaU3* promoter, *Ubiquitin* promoter and zcas9 cassettes (**Figure 7A**). Among the 13 plants tested, four transgene-free homozygous dwarf mutants were obtained (**Figure 7B**). The results demonstrated that the transgene was inherited in a Mendelian fashion, so we could obtain transgene-free dwarf mutants in the F_2_ segregating progeny plants.

To check for potential off-target mutations in transgene-free dwarf maize plants, we searched sites in the maize genome showing high sequence identity to the two sgRNAs used in our study, and identified three potential off-target sites showing two or three nucleotide mismatches with the sgRNA sequences (**Table 1**). The fragments flanking these potential off-target sites were amplified from transgene-free plants and sequenced. The results showed that there was no mutation at the potential off-target sites, which did not have NGG (PAM site) or had more than two different base pairs with sgRNA sequene; For the potential off-target site presenting only a two-base-pair difference from the target site, we also did not detect off-target mutation.

**Table 1:** Evaluation of the off-target effects of CRISPR/Cas9.

Sequence of target site	Sequence of potential off-target sites	Off-target mutation	Loci of the potential off-target sites
**CCC**GGAGCCATTCCTGTGGCCGC	**CTA**GGA**T**CCA**C**T**T**CTGTGGCCGC	NO	Chr8:Intergenic
CTGTCCTTCGGCTTCCACGA**CGG**	CTGTC**G**TTCGGCT**A**CCACGA**CGG**	NO	Chr8:Zm00001d012212 CDS
	CTGT**T**CTT**G**GGCTTCCA**A**GA**AGG**	NO	Chr4:Intergenic

The underlined bold sequence is the PAM. Some letters are indicated in bold to emphasize the difference.

## Discussion

“Green revolution” wheat and rice benefit from the semidwarf phenotype to improve product and lodging resistance (Peng et al., 1999; Sasaki et al., 2002). Although many maize dwarf plants have been reported, few of them have been used for hybrid production. Developing more maize semidwarf mutants and dissecting the function of the causal genes will be helpful for dwarf maize breeding. In this study, we edited the maize *GA20ox3* gene and generated semidwarf maize plants using CRISPR/Cas9 technology. Transgene-free dwarf maize materials were selected and they might be useful for maize breeding in the future.

There are many kinds of gibberellins, which are very important in plant growth and development, mainly in promoting plant internode elongation and leaf growth and breaking seed dormancy (Hedden and Phillips, 2000). GA20-oxidase, which is also known as the “green revolution” gene, belongs to a gene family that contributes to GA biosynthesis. A semidwarf phenotype is also observed in Arabidopsis and rice harboring mutations in GA20 oxidase (Xu et al., 1995; Spielmeyer et al., 2002). Arabidopsis has five GA20ox paralogs, only three of which exhibit GA20ox activity. Arabidopsis *ga20ox1 ga20ox2* double mutant plants exhibit semidwarf and semifertile phenotypes (Rieu et al., 2008), indicating that GA deficiency also influences fertility. Five GA20-oxidase genes have been identified in the maize genome (Song et al., 2011), and we chose *ZmGA20ox3* as the candidate for gene editing due to its high identity with the rice *SD1* gene. The mutant plants in which *GA20ox3* was mutated showed a semidwarf phenotype compared with WT plants. These semidwarf mutant plants will be more useful than severely dwarfed maize materials, because they are more suitable for the heterosis usage in maize production. The semidwarf phenotype of the *ga20ox3* mutants obtained in this study might be due to the functional redundancy of other GA20 oxidases such as GA20ox5. We found that the gene-edited *ga20ox5* mutants also showed a semidwarf phenotype (our unpublished results), indicating that GA20ox5 also has oxidase activity. It might be interesting to investigate whether other maize GA20oxs show such activity.

There are two types of gibberellin-deficient mutants: gibberellin-sensitive and gibberellin-insensitive. The dwarf phenotype of the *ga20ox3* mutant obtained in this study could be recovered by the application of exogenous gibberellin, revealing that it belongs to the gibberellin-sensitive type and confirming that GA20ox3 is involved in GA biosynthesis. In the GA biosynthesis pathway, GA20-oxidase is involved in the successive oxidation steps from GA_53_ to GA_20_ and those from GA_12_ to GA_9_. Consistent with this, the gibberellin content of the *ga20ox3* mutant lacking *GA20-oxidase3* was significantly decreased. To our surprise, GA_19_ content was observed to be unchanged in mut1 plants and to be increased in mut2 plants compared with WT plants. These results indicate that the conversion from GA_44_ to GA_19_ might not be catalyzed by *GA20ox3* but by other *GA20ox* genes. Further experiments should be performed to confirm this hypothesis.

A strict regulatory framework is applied for transgenic crops worldwide. For the gene-edited crops intended for commercial use, it is preferable to eliminate the transgenes from the plants. It has been reported that transgene-free plants with edited genomes can be generated using preassembled CRISPR/Cas9 ribonucleoproteins (Woo et al., 2015). CRISPR/Cas9 ribonucleoproteins (RNPs) also have the advantage of producing transgene-free gene-edited plants (Svitashev et al., 2016; Liang et al., 2017). In genome-edited crops, transgenes and off-targeted genes can also be eliminated by out-crossing or back-crossing (Chen et al., 2018). In our study, transgenes were eliminated by self-crossing, and transgene-free semidwarf mutants were generated. These transgene-free semidwarf mutants could be used for breeding value evaluation without the limitation of regulatory frameworks for transgenic organisms.

## Materials and Methods

### Construction of the sgRNA-Cas9 Expression Vector

Two sgRNA sequences were designed in the first exon of *ZmGA20ox3* (GRMZM2G368411) using CRISPR-P web base resource (http://crispr.hzau.edu.cn/CRISPR2/) (Lei et al., 2014). Two pairs of primers, designated MT1T2-BsF/-BsR and MT1T2-F0/-R0 (**Supplementary Table S1**), were designed according to the two sgRNA sequences. The PCR fragments amplified from pCBC-MT1T2 using the two pairs of primers were inserted between the *Bsa*I sites of pBUE411 (Xing et al., 2014) to construct the pBUE411-2gR-GA vector.

### *Agrobacterium*-Mediated Maize Transformation

The pBUE411-2gR-GA vector was transformed into *Agrobacterium tumefaciens* strain LBA4401. Immature embryos from the maize inbred line Cal were transformed according to the described method (Chen et al., 2018). In brief, 1–1.2 mm immature embryos were isolated and suspended in liquid infection medium. The embryos were heated at 45°C for 3 min and then transferred to an *A. tumefaciens* suspension and incubated for an additional 5 min. The embryos were then transferred to solid cocultivation medium and incubated in the dark at 23°C for 3 days. The embryos were subsequently transferred to resting medium and cultured at 28°C for 7 days. The embryos were next transferred to selection medium and maintained for 2 weeks under dim light (10 μmol m^-2^ s^-1^) at 28°C. Resistant calli were regenerated under fluorescent white light under a 16/8 h light/dark cycle. The regenerated shoots were transferred to rooting medium. Two weeks later, the regenerated T_0_ seedlings were transferred to soil and grown in a greenhouse with a 16/8 h light/dark cycle at 25–28°C.

### Maize Propagation

T_1_ generation plants were produced by crossing the transgenic T_0_ plants with the maize inbred line Cal. These T_1_ plants were self-pollinated to produce the T_2_ generation. The T_1_ and T_2_ plants were grown in the field or in a greenhouse in Beijing, China.

### PCR Analysis of the Transgenic Plants

Maize genomic DNA was extracted from the leaves using the cetyltrimethylammonium bromide (CTAB) method (Murray and Thompson, 1980). To determine the mutations in the target gene, fragments flanking the target site were amplified by PCR using genomic DNA as the template. For the transgenic T_0_ and T_1_ generation plants, PCR products were cloned into pEASYR-Blunt Simple Cloning vectors (TransGen, Beijing, China), six randomly selected individual clones were sequenced, or the PCR products were directly sequenced. For the transgenic T_2_ generation plants, the PCR products were directly sequenced. For the determination of transgene-free plants, several fragments from the T-DNA region of the pBUE411-2gR-GA vector were amplified by PCR with genomic DNA as the template. The primers are listed in **Supplementary Table S1**.

### Endogenous GAs Determination

The shoot tips of 7-week-old homozygous mutant and WT plants grown in greenhouse were sampled, and each sample was collected from 13–15 plants. The samples were sent to the Core Facility for Hormone Detection and Analysis (Institute of Genetics and Developmental Biology, Chinese Academy of Sciences, Beijing, China) for endogenous GAs determination using UPLC-MS/MS, according to the described method (Ma et al., 2015).

### GA_3_ Application in the Mutant Plants

Six week after seedling emergence, the plants were sprayed with 100 mg/L GA_3_ three times per week and treated for another three weeks. As the GA_3_ stock was dissolved in 20% ethanol, the control plants were sprayed with 20% ethanol. The heights of the seedlings and plants were measured from the soil surface to the highest point of the seedlings or plants once a week. Plant height was measured every week from the ninth week after seedling emergence onward.

### Quantitative Real-Time PCR Analysis

Two week after seedling emergence, the plants were sprayed with 100 mg/L GA_3_ one times per day. Shoot samples were collected one week later for total RNA isolation. The first-strand cDNA synthesis was performed with the M-MuLV reverse transcriptase (Promega) using total RNA as template. For the quantitative real-time PCR (qRT-PCR), 1 μL of cDNA was mixed with 2× SYBR premix ExTaq (Takara), 0.2 μM forward primer, 0.2 μM reverse primer and 0.4 μL 50× ROX in 20 μL of reaction mixture. qRT-PCR was conducted by the ABI 7300 system using the following protocol: 95 °C for 2 min, 40 cycles at 95 °C for 5 s, 58 °C for 30 s, and 72 °C for 31 s. The relative transcriptional levels were calculated using the 2^-ΔΔ^ *^C^* ^t^ method with *GAPDH* as a housekeeping gene.

### Scanning Electron Microscopy

The middle part of the stem at the spike position was harvested when the plants had grown to the tasseling stage. Cross-sections and longitudinal sections were prepared, immediately immobilized in a 2% glutaraldehyde solution, and fixed for 48 hours without light at room temperature. The materials were washed three times with phosphoric acid buffer for 30 min each time and then dehydrated in 30%, 50%, 70%, 80%, 90%, and 100% alcohol. The sections were observed using an Olympus SZX7 stereomicroscope.

The cell numbers within the 0.81 mm^2^ area were counted and the area of each cell was calculated with the formula: 0.81 mm^2^/cell numbers. The cell numbers within 1 mm distance were counted and the cell length was calculated with the formula: 1 mm/cell numbers.

### Data Treatment

Comparisons of values for significant differences were made using Student’s *t* test in Excel (Microsoft).

## Data Availability Statement

The datasets generated for this study are available on request to the corresponding authors.

## Author Contributions

YJL, ZR and GW designed the research. JZ performed the major experiments. XZ, RC, LY, KF, YL, and ZR collected the samples and analyzed the data. YJL, ZR, and JZ wrote the article.

## Funding

The research was supported by the Fundamental Research Funds for Central Non-Profit of Institute of Crop Sciences, Chinese Academy of Agricultural Sciences (S2018QY07) and the National Major Project for Transgenic Organism Breeding (2016ZX08009003-004, 2016ZX08010-004).

## Conflict of Interest

The authors declare that the research was conducted in the absence of any commercial or financial relationships that could be construed as a potential conflict of interest.
