# Supplementary material for: Generation of Transgene-Free Semidwarf Maize Plants by Gene Editing of Gibberellin-Oxidase20-3 Using CRISPR/Cas9
Source: Front Plant Sci. 2020 Jul 9;11:1048. doi: 10.3389/fpls.2020.01048 (PMC7365143; doi:10.3389/fpls.2020.01048)
Supplement: Supplementary file 1 [file DataSheet_1.docx]

**Supplementary Table S1 Primers used for vector construct and PCR analysis**

| primer name | Primer Sequence (5’-3’) | Function |
| --- | --- | --- |
| Target1 | GGAGCCATTCCTGTGGCCGC | The target site |
| Target2 | CTGTCCTTCGGCTTCCACGA | The target site |
| MT1-BsF | AATAATGGTCTCTGGCGGCGGCCACAGGAATGGCTCCGTT | amplify the pCBC-MT1T2  fragment |
| MT1-F0 | TGGCGGCCACAGGAATGGCTCCGTTTTAGAGCTAGAAATAGC | amplify the pCBC-MT1T2  fragment |
| MT2-R0 | AACTCGTGGAAGCCGAAGGACAG CGCTTCTTGGTGCC | amplify the pCBC-MT1T2  fragment |
| MT2-BsR | ATTATTGGTCTCTAAACTCGTGGAAGCCGAAGGACAGC | amplify the pCBC-MT1T2  fragment |
| 1F | GACAGGCGTCTTCTACTGGTGCTAC | Amplify OsU3 terminator to TaU3 promoter fragment |
| 1R | CTCACAAATTATCAGCACGCTAGTC | Amplify OsU3 terminator to TaU3 promoter fragment |
| 2F | GCCAGTGTTTCTCTTTGGGG | Amplify Ubiquitin promoter and zCas9 fragment |
| 2R | CATCGCTATTGTCCGGGTTG | Amplify Ubiquitin promoter and zCas9 fragment |
| 3F | TGCCTACCACGAGAAGTACC | Amplify zCas9 fragment |
| 3R | TAGAAGTCCTCCTGCCTCCT | Amplify zCas9 fragment |
| GA20-3-T1-1-2F | CTGTTCCATGCAGCGTTTT | Amplify and sequence the off-target flanking fragment |
| GA20-3-T1-1-2R | ACATCCCTGTCCCTTCTGAA | Amplify and sequence the off-target flanking fragment |
| GA20-3-T2-1-4F | GAGGTGCCGATGGTGGAC | Amplify and sequence the off-target flanking fragment |
| GA20-3-T2-1-4R | CTCCTTCATCTCCTCGCAGT | Amplify and sequence the off-target flanking fragment |
| GA20-3-T2-2-2F | CGCTAGAAGAGTATGGCAGC | Amplify and sequence the off-target flanking fragment |
| GA20-3-T2-2-2R | AGCGATCAGATCCAGAGCAA | Amplify and sequence the off-target flanking fragment |

**Supplemental Table S2. Primers used for the qRT-PCR**

| Primers | Primer Sequences (5’-3’) | Gene ID |
| --- | --- | --- |
| ZmKO1-F | ACTTGGCTGGCTATGAGGTT | Zm00001d046344 |
| ZmKO1-R | TCAAACCTCCCGTCCAGAAA |  |
| ZmKO2-F | CATCCAGGCAGTGAACATCG | Zm00001d046342 |
| ZmKO2-R | AGGTACACATGCAACGGGTA |  |
| GA20ox3-F | TCCCTGCACCTAACGAGC | Zm00001d042611 |
| GA20ox3-R | ACATGTCTGCCACCTGATCA |  |
| GA20ox5-F | CCGTCTCTTTCGCCCCAC | Zm00001d012212 |
| GA20ox5-R | AGTAGGGCCAGCGTCAAAA |  |
| GA20ox1-F | CATCCCGCAGCAGTTCATC | Zm00001d034898 |
| GA20ox1-R | CGTGAAGAAGGCGTCCATG |  |
| Dwarf8-F | GACACCGTGCACTACAATCC | Zm00001d033680 |
| Dwarf8-R | GGAGATCGAAGTAGCCAGCA |  |
| GID1-F | AGCAGCACATGCACACAAAC | Zm00001d038165 |
| GID1-R | GCAACCCGACGACGAAAC |  |
| GA2ox1-F | GAGCAAGCTAAGGATCTGCG | Zm00001d037565 |
| GA2ox1-R | CGACTCACTCCTGCGGATAA |  |
